# Protective Effect of Resveratrol on Biomarkers of Oxidative Stress Induced by Iron/Ascorbate in Mouse Spermatozoa

**DOI:** 10.3390/nu6020489

**Published:** 2014-01-27

**Authors:** María Angélica Mojica-Villegas, Jeannett Alejandra Izquierdo-Vega, Germán Chamorro-Cevallos, Manuel Sánchez-Gutiérrez

**Affiliations:** 1 Preclinical Toxicology Laboratory, Department of Pharmacy, National School of Biological Sciences, National Polytechnic Institute, Mexico City 07510, Mexico; E-Mails: moviangel13@yahoo.com.mx (M.A.M.-V.); gchamcev@yahoo.com.mx (G.C.-C.); 2 Academic Area of Medicine, Institute of Health Sciences at Autonomous University of the State of Hidalgo, Pachuca, Hidalgo 11510, Mexico; E-Mail: jizquierdovega@gmail

**Keywords:** resveratrol, spermatozoa, oxidative stress, *in vitro* fertilization, mitochondrial transmembrane potential

## Abstract

Resveratrol (RVT) is a polyphenolic compound found mainly in the grape and attributed with various pharmacological properties, among them their antioxidant activity. In the present study, we assess the antioxidant activity of resveratrol on oxidative damage induced by ferrous iron/ascorbate (100 µM/150 µM) in sperm of CD1^+^ mice. We evaluated several parameters in spermatozoa treated with or without resveratrol: (i) sperm quality analysis; (ii) mitochondrial transmembrane potential (Δψ_m_); (iii) ROS generation; (iv) superoxide dismutase (SOD) activity; (v) glutathione peroxidase (GPX) activity; (vi) lipid peroxidation; (vii) and *in vitro* fertilization (IVF) capability. Spermatozoa treated with RVT (15 µg/mL) before ferrous iron/ascorbate treatment exhibited: a significant increase in motility (8-fold), a significant increase in viability (2-fold), a significant increase in Δψ_m_ (1.15-fold), accompanied with a significant decrease in the generation of ROS (4.96-fold), a significant decrease in GPX activity (1.32-fold), and a significant decrease in lipid peroxidation concentration (10.29-fold) relative to spermatozoa treated with ferrous iron/ascorbate; however, no changes in SOD activity were observed. Finally, spermatozoa treated with RVT before ferrous iron/ascorbate treatment showed a significant increase in oocyte fertilization (1.2-fold), relative to spermatozoa treated with ferrous iron/ascorbate. These results suggest that RVT possesses antioxidant properties that may prevent the deleterious effects produced by oxidative damage on spermatozoa, resulting in the maintenance of fertility.

## 1. Introduction

Oxidative stress is defined as an imbalance between the formation of reactive oxygen species (ROS, including hydroxyl radicals, superoxide anions and hydrogen peroxide) and the antioxidant capacity [1]. Spermatozoa are particularly susceptible to oxidative stress due to the high membrane content of polyunsaturated fatty acid and the intracellular deficiency of antioxidant enzymes [2].

Low ROS levels are required for the regulation of the principal functions of sperm. Such functions are capacitation, acrosome reaction and fertilizing ability [3]. However, when levels are excessive, ROS attack polyunsaturated fatty acids in the sperm plasma membrane, leading to lipid peroxidation [4]. An imbalance between ROS generation and the antioxidant capacity of spermatozoa leads to oxidative stress, and the exposure of spermatozoa to this condition appears to be related to male infertility [3,5].

Seminal plasma and sperm are endowed with an array of antioxidant enzymes that act as free radical scavengers to protect spermatozoa against cellular damage caused by oxidative stress [6]. Such enzymes include glutathione peroxidase, glutathione reductase, superoxide dismutase, catalase, low molecular weight antioxidants, vitamin E, and vitamin C. Numerous attempts have been made to avoid or minimize oxidative stress in sperm by the use of antioxidants. However, the effectiveness of this strategy is still being debated [6,7].

A great variety of substances are antioxidants, including vitamins, enzymes and other free radical scavengers. Resveratrol (RVT) (trans-3,5,4′-trihydroxystilbene) is a polyphenol compound present in grapes, peanuts, berries and wine [8]. It is a phytoalexin whose biological function is to protect the plant in case of parasitic attack or environmental stress [9]. The literature contains numerous reports on the wide variety of properties of RVT, including its anti-inflammatory, cardioprotective, anticancer, antimicrobial, anti-aging and antioxidant effects [10]. This biological activity is carried out by a wide variety of mechanisms, one of the most important of which is antioxidant activity due to free radical scavenging [11]. Some recent studies in animal models demonstrate that RVT has positive effects on the hypothalamic-pituitary-gonad axis, blood testosterone levels, sperm production and sperm motility [12,13]. Furthermore, RVT may decrease germ cell apoptosis [14,15]. Despite these insights, the effect of RVT supplementation on male infertility has not yet been explored.

The aim of the present study was to explore the antioxidant activity of RVT against oxidative stress induced in mouse spermatozoa by exposure to ferrous iron/ascorbate *in vitro*.

## 2. Experimental Section

### 2.1. Chemicals

Resveratrol, bovine serum albumin fraction V (BSA), butylated hydroxytoluene (BHT), human chorionic gonadotropin (hCG), lactic acid, sodium pyruvate, formaldehyde, thiobarbituric acid (TBA) and trypan blue were purchased from Sigma Chemical Co. (St Louis, MO, USA). Gonadotropin (PMSG) from pregnant mare serum was acquired from Intervet, International B.V. (Boxmeer, Holland). The mito Probe JC-1 assay kit and an ROS detection reagent were from Molecular Probes Invitrogen (Mount Waverley, Australia), trichloroacetic acid (TCA) from J.T. Baker (Pillipsburg, NJ, USA), and the RANSOD and RANSEL kits from RANDOX (Crumlin, UK).

### 2.2. Animals

Animals were maintained according to the norms of the Institutional Ethics Animal Care and Use Committee (CIECUAL), and in compliance with the Guidelines for Use and Care of Laboratory Animals. Male sexually mature (30 ± 2 g) and female immature CD1^+^ mice (5 old weeks) were obtained from the Institute of Health Sciences at the Autonomous University of Hidalgo (Hidalgo, Mexico). Animals were maintained under standard conditions, with a 12 h/12 h light/dark cycle, constant temperature (22 ± 2 °C) and humidity (50%), and food and water freely available in their home cages.

### 2.3. Experimental Design

After animal sacrifice by cervical dislocation, testes-epididymis-vas deferent complexes were dissected. Spermatozoa were collected by flushing vas deferens and cauda epididymis lumens with 1 mL of Media M-16 (100 mM NaCl, 25 mM NaHCO_3_, 5.5 mM glucose, 2.6 mM KCl, 1.56 mM Na_2_HPO_4_, 0.5 mM sodium pyruvate, 1.8 mM CaCl_2_, 0.5 mM, MgCl_2_ and 20 mM sodium lactate, pH 7.4) at 37 °C. All medium were prepared with deionized water (Milli-Q Plus water system, Millipore). The spermatozoa were allowed to swim in the medium for 10 min at 37 °C. The sperm suspension was then transferred into a plastic tube and the motility and concentration of sperm were determined. Samples were used only if at least 80% of total sperm were motile and the concentration was at least 10 × 10^6^ sperm/mL.

The sperm suspensions from three animals were pooled and divided into four experimental groups: (1) spermatozoa without treatment (Control); (2) spermatozoa treated with 15 µg/mL RVT during 15 min; (3) spermatozoa treated with 100 µM/150 µM ferrous iron/ascorbate for 45 min; and (4) spermatozoa treated with 15 µg/mL RVT during 15 min before treatment with 100 µM/150 µM ferrous iron/ascorbate for 45 min. After incubation, the following parameters were evaluated in the four groups: (i) sperm quality; (ii) mitochondrial transmembrane potential (Δψ_m_); (iii) ROS generation; (iv) superoxide dismutase activity (SOD); (v) glutathione peroxidase (GPX) activity; (vi) lipid peroxidation; and (vii) *in vitro* fertilization (IVF) capability. For each parameter, three independent experiments were performed in duplicate.

### 2.4. Spermatozoa Quality

Sperm parameters, including viability and motility, were evaluated according to a previously described method [16]. Spermatozoa motility (percent motile cells) was assessed in ten random fields using a phase contrast microscope (Carl Zeiss, Germany) at 400X. Sperm concentration was measured in a hemocytometer and expressed as one million/mL of suspension. Spermatozoa viability was determined by trypan blue exclusion assay. For each sample, 100–200 cells were counted.

### 2.5. Mitochondrial Membrane Potential (Δψ_m_)

Spermatozoa Δψ_m_ was measured using a spectrofluorometer (Perkin Elmer LS 55, Norwalk, CT, USA) and a MitoProbe JC-1 assay kit. JC-1 is a lipophilic cation that differentially labels mitochondria on the basis of membrane potential. After incubating a solution of 5 × 10^6^ spermatozoa/mL with one of the treatments, two micro liters of JC-1 were added. High membrane potential was associated with emission at 590 nm (red), and low membrane potential with emission at 530 nm (green) when spermatozoa were excited at 488 nm. The Δψm was determined by a ratio of fluorescence intensity at 590 nm and at 530 nm.

### 2.6. ROS Level in Spermatozoa

ROS generation was evaluated using (5-(and-6)-chloromethyl-2′,7′-dichlorofluorescein diacetate and acetyl ester (CM-H_2_DCFDA). The method is based on the ROS-dependent oxidation of 2′,7′-dichlorofluorescin diacetate to fluorescent dichlorofluorescein (Invitrogen Molecular Probes). Spermatozoa (5 × 10^6^/mL) were treated with 10 µM CM-H_2_DCFDA for 30 min at 37 °C in the dark. Fluorescence intensities were measured using a spectrofluorometer (Perkin Elmer LS 55, Norwalk, CT, USA).

### 2.7. SOD Activity in Spermatozoa

Enzymatic activity of SOD was measured using the RANSOD assay kit (RANDOX). This method uses xanthine oxidase to generate superoxide radicals, which react with 2-(4-iodophenil)-3-(4-nitrophenol)-5-phenyltetrazolium chloride to form a red formazan dye. SOD activity was measured at 505 nm with a spectrophotometer (Power Wave Xs, Biotek Instruments Inc., Highland Park, Winooski, VT, USA), based on evaluating the degree of inhibition of xanthine to water and molecular oxygen. 10 × 10^6^ Cells were treated with 1:1 0.1% triton X 100-PBS for 20 min. The cells were then centrifuged at 5000 rpm at 4 °C for 10 min. Finally, the supernatant was obtained for determining SOD activity.

### 2.8. GPX Activity in Spermatozoa

Enzymatic activity of GPX was measured using the RANSEL assay kit (RANDOX). GPX catalyzes glutathione oxidation (GSH) by cumene hidroperoxide. Oxidized glutathione (GSSG) in the presence of glutathione reductase (GR) and NADPH is immediately changed to the reduced form with a concomitant oxidation of NADP^+^. GPX activity was measured as the decrease of absorbance at 340 nm with a spectrophotometer (Power Wave Xs, Biotek Instruments Inc., Highland Park, Winooski, VT, USA). 10 × 10^6^ Cells were treated with 1:1 0.1% triton X 100-PBS for 20 min. The cells were then centrifuged at 5000 rpm at 4 °C for 10 min. Finally, the supernatant was obtained for determining GPX activity.

### 2.9. TBARS Concentration in Spermatozoa

Malondialdehyde (MDA), other aldehydes and lipid hydroxyperoxides are able to form adducts with TBA (Sigma-Aldrich Co., St. Louis, MO, USA). The MDA concentration was used as an index of lipid peroxidation using the thiobarbituric acid reactive substances (TBARS) method [17]. Briefly, sperm suspensions (10 × 10^6^ cells/mL) were added to a mixture containing 0.5% TBA and 3.75% BHT in methanol. Samples were heated in a boiling water bath for 30 min and then cooled. Absorbance was then measured at 532 nm with a spectrophotometer (Power Wave Xs, Biotek Instruments Inc., Winooski, VT, USA).

### 2.10. *In Vitro* Fertilization (IVF)

#### 2.10.1. Egg Recovery

Eggs were obtained from immature female CD1^+^ mice. Super ovulation was induced by intraperitoneal injection of 10 IU Pregnant mare’s serum gonadotropin (PMSG), and 48 h later of 10 IU human chorionic gonadotropin (hCG). Approximately 14–16 h after the hCG injection, animals were sacrificed by cervical dislocation. The uterine ovary-salpinge-horn complex was dissected and suspended in M-16 medium. In each oviduct, the ampulla were punctured, and the cumulus-egg complex was extruded and placed in a solution of 0.1% (w/v) hyaluronidase with M-16 medium for seven min at 37 °C to remove cumulus cells. Then, cumulus free eggs were pooled and washed with M-16 medium to remove hyaluronidase. The fertilization assay was carried out only with mature eggs showing a polar body and with the zone intact.

#### 2.10.2. IVF Assay

For each experimental group 40 random eggs in 200 µL M-16 medium were placed on a standard slide with two polished spherical depressions of approximately 0.5 to 0.8 mm deep (VWR International). The eggs were then inseminated with 10 µL (1 × 10^5^ cells) of one of four sperm suspensions (see Section 2.3). In each case, the sperm suspension had been previously capacitated in M-16 medium (supplemented with 4 mg/mL BSA) for 1 h at 37 °C. Gametes were co-incubated during 24 h at 37 °C in a high-humidity incubator under an atmosphere of 5% CO_2_/95% air. The embryos were examined for the presence of two to six cells as an indication of successful fertilization. The cells were fixed in 3% formaldehyde-PBS (v/v) and observed by phase contrast microscopy. Three independent experiments with different batches of cells were performed.

### 2.11. Data Analysis

Statistical analysis was carried out with analyses of variance (ANOVA) followed by a Bonferonni correction to evaluate pair wise differences. A *p*-value < 0.05 was considered significant. Data are expressed as the means ± standard deviation (SD). All analyses were performed using the statistical software Stata 8.0 (Stata Corp., College Station, TX, USA).

## 3. Results

### 3.1. Effect of RVT on Spermatozoa Quality

Ferrous iron/ascorbate (100 µM/150 µM) was used to induce oxidative stress and cause deleterious effects on spermatozoa quality. The results show a significant reduction (*p* < 0.001) in motility (11.0 fold) and viability (4.39 fold). On the other hand, pretreatment with 15 µg/mL of RVT for 15 min provided protection of spermatozoa. Compared to the spermatozoa treated only with iron/ascorbate, the RVT pretreated spermatozoa showed an 8.0 fold increase in motility and a 2.0 fold increase in viability (Table 1; *p* < 0.001). Regarding both parameters, there was a significant enhancement of spermatozoa quality. 

**Table 1:** Spermatozoa quality.

Sperm parameters	Control	RVT	Ferrous iron/ascorbate	RVT + Ferrous iron/ascorbate
Motility (%)	73.3 ± 5.7	73.3 ± 2.8	6.66 ± 2.8 *	53.3 ± 5.7 **
Viability (%)	65.0 ± 8.6	59.6 ± 6.1	14.8 ± 3.3 *	30.5 ± 6.5 **

Values represent the mean ± SD; * *p* < 0.001 *vs.* control group; ** *p* < 0.001 *vs.* Fe^2^/asc group.

### 3.2. Protective Effect of Resveratrol on Mitochondrial Function

**Figure 1:**
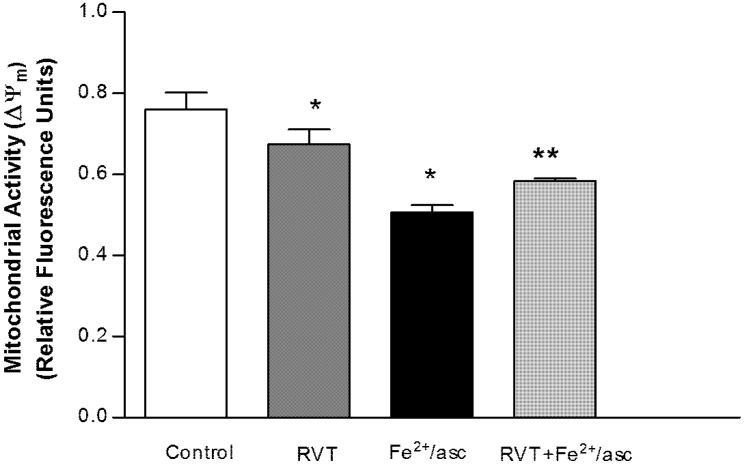
Effect of RVT on Δψ_m_. Spermatozoa were obtained from ductus deferens in M-16 medium, pooled and incubated with RVT, Fe^2+^/asc or RVT + Fe^2+^/asc. Δψ_m_ was determined using the Mito ProbeJC-1 assay kit and the fluorescence was measured on a spectrofluorometer. Values represent the mean ± SD of three independent experiments performed in duplicate. * *p* < 0.05 *vs.* control group. ** *p* < 0.05 *vs.* Fe^2+^/asc group.

The evaluation of mitochondrial membrane potential (Δψ_m_) is a reliable indicator of sperm quality. We evaluated mitochondrial activity using fluorophore JC-1. Compared with the control group, the ferrous iron/ascorbate treatment significantly reduced the Δψ_m_ of spermatozoa (1.5 fold). Contrarily, compared to treatment with ferrous iron/ascorbate alone, pretreatment with RVT followed by exposure to ferrous iron/ascorbate resulted in spermatozoa with a higher Δψ_m_ (1.15 fold; *p* < 0.05; Figure 1). Interestingly, spermatozoa treated only with RVT showed a significant decrease in the Δψ_m_ (1.13 fold) compared with the control group.

### 3.3. Resveratrol Diminished Intracellular ROS Generation

**Figure 2:**
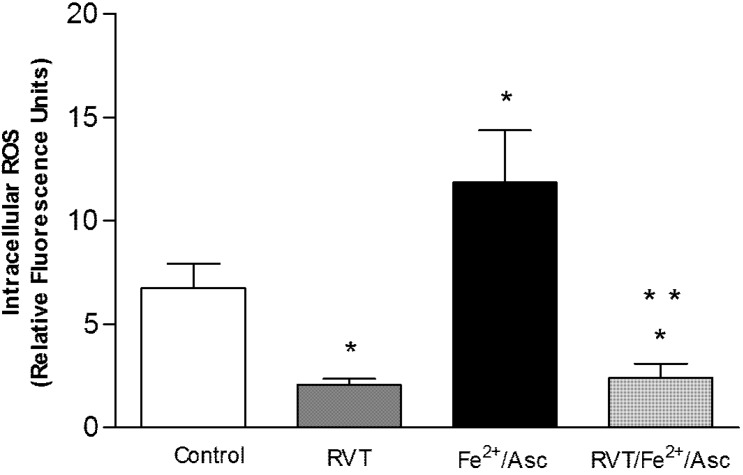
Effect of RVT treatment on intracellular ROS levels. Spermatozoa were obtained from ductus deferens in M-16 medium, pooled and incubated with RVT, Fe^2+^/asc or RVT + Fe^2+^/asc. ROS levels were evaluated using the CM-H_2_DCFDA reagent. Fluorescence was measured in a spectrofluorometer. Values represent the mean ± SD of three independent experiments performed in duplicate. * *p* < 0.001 *vs.* control group. ** *p* < 0.001 *vs.* Fe^2+^/asc group.

ROS generation was measured by fluorescence using the reactive oxygen species detection reagent. Compared with the control, there was a significantly greater level of ROS (1.75 fold) in spermatozoa treated only with ferrous iron/ascorbate (Figure 2). On the other hand, compared to spermatozoa treated only with ferrous iron/ascorbate, spermatozoa pretreated with RVT and then exposed to ferrous iron/ascorbate had a significant lower level of ROS (4.96 fold; *p* < 0.001).

### 3.4. Effect of RVT on SOD and GPX Activity

**Figure 3:**
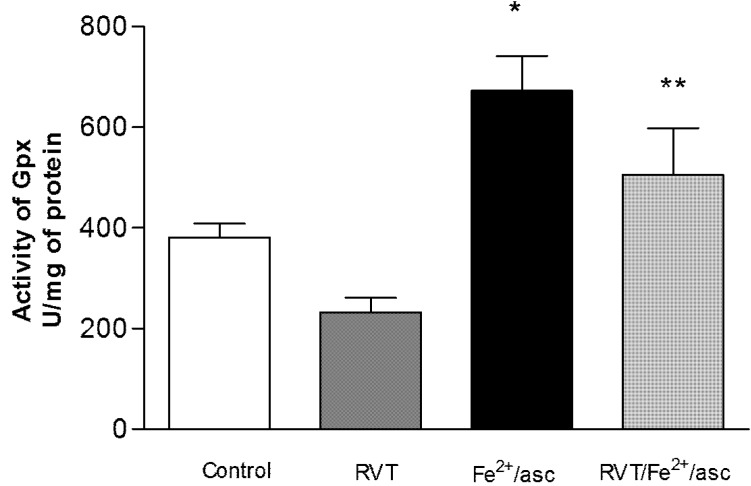
Effect of RVT on GPX activity. Spermatozoa were obtained from ductus deferens in M-16 medium, pooled and incubated with RVT, Fe^2+^/asc or RVT + Fe^2+^/asc. Activity of GPX was measured with a spectrophotometer after using the RANSEL assay kit. Values represent the mean ± SD of three independent experiments performed in duplicate. * *p* < 0.05 *vs.* control group. ** *p* < 0.05 *vs.* Fe^2+^/asc group.

**Figure 4:**
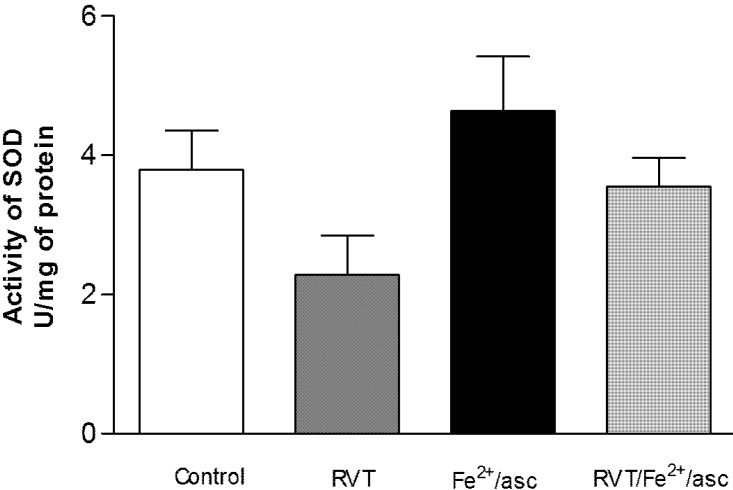
Effect of RVT on SOD activity. Spermatozoa were obtained from ductus deferens in M-16 medium, pooled and incubated with RVT, Fe^2+^/asc or RVT + Fe^2+^/asc. Enzymatic activity of SOD was measured with a spectrophotometer after using the RANSOD assay kit. Values represent the mean ± SD of three independent experiments performed in duplicate. No significant differences were observed.

SOD and GPX activity was analyzed in the spermatozoa of all treatments groups to evaluate oxidative stress. Compared with the control, treatment only with ferrous iron/ascorbate led to a significantly greater GPX activity in spermatozoa (1.76 fold; Figure 3). Contrarily, compared to treatment only with ferrous iron/ascorbate, pretreatment with RVT followed by exposure to ferrous iron/ascorbate resulted in significantly lower GPX activity (1.32 fold). No significant differences were found in SOD activity between any of the groups (Figure 4).

### 3.5. Effect of RVT on Lipid Peroxidation

**Figure 5:**
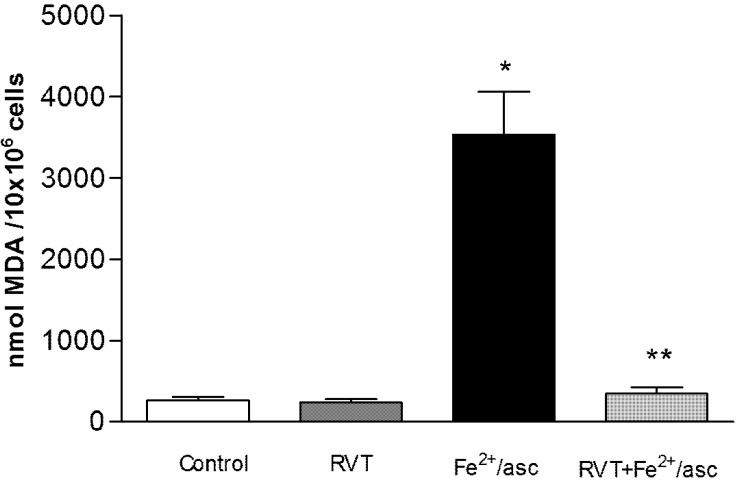
Effect of RVT on lipid peroxidation. Spermatozoa were obtained from ductus deferens in M-16 medium, pooled and incubated with RVT, Fe^2+^/asc or RVT + Fe^2+^/asc. Lipid peroxidation was measured with a spectrophotometer using the TBARS method. Values are the mean ± SD of three independent experiments performed in duplicate. * *p* < 0.001 *vs.* control group. ** *p* < 0.001 *vs.* Fe^2+^/asc group.

Lipid peroxidation was assessed as a marker of oxidative damage (Figure 5). Compared to the control, spermatozoa treated only with ferrous iron/ascorbate showed a significantly higher content of MDA (13.38 fold). In contrast, compared to treatment only with ferrous iron/ascorbate, pretreatment with RVT followed by exposure to ferrous iron/ascorbate resulted in a significant reduction in the MDA content (10.29 fold) of spermatozoa (*p* < 0.001).

### 3.6. Resveratrol Retains in Vitro Fertilization Capability

**Figure 6:**
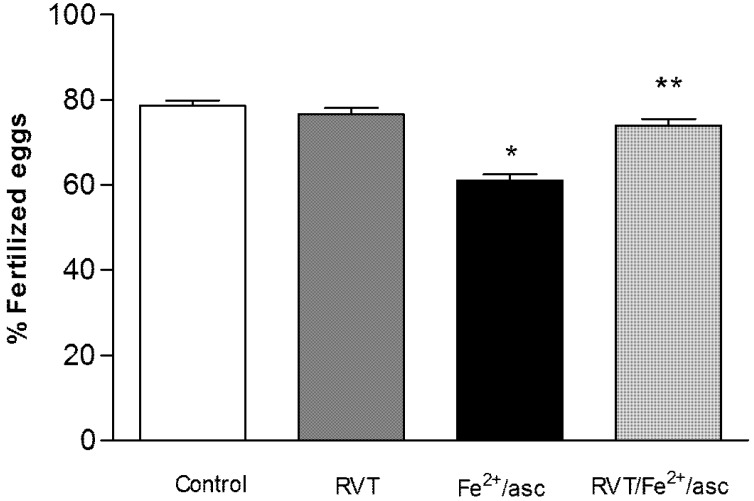
Effect of RVT on IVF capability. Spermatozoa from the different groups were incubated with eggs during 24 h at 37 °C in an incubator under an atmosphere of 5% CO_2_/95% air. Eggs with 2 to 6 cells were considered fertilized. Values represent the mean ± SD of three independent experiments performed in duplicate. * *p* < 0.001 *vs.* control group. ** *p* < 0.001 *vs.* Fe^2+^/asc group.

Compared to untreated (control) and RVT-only treated spermatozoa, a significantly lower percentage of these cells treated only with ferrous iron/ascorbate had the capacity to fertilize oocytes (78.66% and 76.66% *vs.* 61%, respectively; Figure 6). On the other hand, compared to treatment only with ferrous iron/ascorbate, pretreatment with RVT followed by exposure to ferrous iron/ascorbate resulted in a significantly greater percentage of spermatozoa with the capacity to fertilize oocytes (74% *vs.* 61%; *p* < 0.001).

## 4. Discussion

Oxidative stress is defined as an imbalance between ROS generation and antioxidant capacity. As mentioned previously, spermatozoa are highly vulnerable to attack from ROS due to their low cytoplasmic antioxidant capacity and high membrane polyunsaturated fatty acid content [18].

Whereas the generation of low levels of ROS is an important component of the signal transduction stimulating capacity of spermatozoa [19], excessive ROS levels induce lipid peroxidation and reduce the ionophore-induced acrosome reaction [20]. Only acrosome-reacted sperm are able to penetrate the pellucida zone of the oocyte [21].

The possible relationship of oxidative stress in spermatozoa to male infertility is supported by both experimental and epidemiological evidence [3,5]. High ROS concentrations lead to pathological changes in spermatozoa by intensifying the peroxidation of lipids found in the plasma membrane of these cells, which in turn impairs the fusion of the plasma of the male and female gametes [3]. Thus excessive ROS levels affect basic parameters of sperm, such as sperm count, morphology and motility [22]. ROS is also able to stimulate the oxidation of the sulfhydryl radical as well as the DNA of molecules. Moreover, DNA fragmentation (an indicator of nuclear structural integrity) was shown to correlate strongly with ROS concentration [23]. Further evidence of the damage caused to spermatozoa by excessive levels of ROS is provided by the fact that levels of DNA oxidation in sperm are higher in infertile than fertile men [24].

The use of antioxidant supplementation with vitamins E and C, GSH, catalase, selenium or SOD has been a strategy for the treatment of oxidative stress. However, results have caused controversy over the effectiveness of this strategy for improving fertility [25,26]. Recently, some studies have reported a dose-response relationship between the daily intake of antioxidant nutrients (carotenoids, lycopene and lutein) and semen quality [27,28]. In addition, an inverse relationship has been shown between the intake of whole dairy foods (with the fat content intact) and semen quality in young men [29].

Consequently, there is great interest in evaluating antioxidant agents that could protect spermatozoa from ROS. One possible agent, resveratrol (RVT), is a natural phytoalexin with antioxidant properties. It is widely consumed in the Mediterranean diet in the form of peanuts, grapes and wine [8]. Assays with *in vivo* experimental models have shown a positive RVT-induced effect on male reproduction, reported as the enhancement of testosterone levels, motility and sperm count [13]. Moreover, it has recently been shown that *in vivo* treatment with RVT prevents oxidative stress in testes of hyperthyroid rats [30] and rats treated with a chemotherapy drug [31]. However, the antioxidant mechanism of action of RVT on male reproduction is as yet unknown. The aim of the present study was to carry out an *in vitro* analysis of the protective effect of RVT against oxidative damage caused by ferrous iron/ascorbate on mouse spermatozoa. 

The daily intake of RVT in the Mediterranean diet is approximately 0.02 mg/kg, assuming that red wine is the main dietary source and the average concentration of trans-RVT in wine is 5 mg/L [32,33]. Recent *in vivo* studies have shown that repeated doses of RVT at 10 mg/kg, 20 mg/kg and 50 mg/kg, being 500, 1000, 2500 times higher than the aforementioned average consumption, respectively, provides a sufficiently large safety margin [34] and protects testes and spermatogenesis against oxidative stress [13,30,31]. Unfortunately, RVT concentrations in testes of animals receiving chronic treatment with this polyphenol are unknown. Additionally, the low plasmatic levels of RVT attained (<40 nM) in such animals would be inadequate for eliciting a biological effect, considering the quantity needed for activity in cells with *in vitro* concentrations of 5–100 µM [35]. Based on an evaluation of motility of spermatozoa with different concentrations of RVT (data not shown), 15 µg/mL was the dose of this compound chosen for the present study. In accordance with this decision, an antioxidant effect on spermatozoa has been found with RSV treatment when employing concentrations in the range 5 to 20 µg/mL [36].

Several studies have demonstrated that ferrous iron/ascorbate causes oxidative damage in the spermatozoa of various animal species [37,38]. The ferrous iron/ascorbate system generates the hydroxyl radical through the Haber-Weiss or Fenton reaction [39]. High levels of ROS are associated with a reduction in motility and ATP levels of spermatozoa, which is attributed to mechanisms independent of oxidative phosphorylation that can lead to the inactivation of glycolytic enzymes [40]. In the present study we observed that the diminution in quality (motility and viability) of sperm induced by the pro-oxidant ferrous iron/ascorbate was prevented by RVT, suggesting antioxidant activity by the latter compound. There is evidence that RVT is an effective scavenger of the hydroxyl radical and superoxide anion, as well as other radicals induced by metals [41].

Spermatozoon motility has been associated with the functional status of mitochondria because motility is ATP-dependent. Since mitochondrial membrane potential (Δψ_m_) is widely used to assess the functional status of mitochondria [42], we evaluated this parameter in spermatozoa. Results show that RVT pretreatment protected against the decrease in Δψ_m_ caused when spermatozoa are treated only with ferrous iron/ascorbate. Additionally, we found that treatment only with RVT significantly decreased Δψ_m_ without any change in motility. Consistent with this observation, it has been shown that Δψ_m_ decreased in a concentration-dependent manner with increasing concentrations of RVT (including the same concentration used in this study), without affecting motility, acrosome integrity or plasma membrane integrity in ram spermatozoa [36]. Likewise, a decrease in the Δψ_m_ of neuroblastoma cells was found as a result of RVT treatment in a concentration-dependent manner [43]. 

Concerning the effects of RVT on mitochondrial bioenergetics, it has been shown that this compound inhibits the brain mitochondrial respiratory chain of complex III, thus preventing the production and inducing the scavenging of ROS [44]. The present study shows that RVT prevents the generation of intracellular ROS induced by the pro-oxidant ferrous iron/ascorbate in spermatozoa, suggesting the antioxidant property of RVT as an ROS scavenger. This protective role is further evidenced by the enzymatic activity of GPx in spermatozoa. GPx catalyzes the reduction of peroxide by oxidizing glutathione. We found that RVT prevents the increase in GPx activity that results from exposure to ferrous iron/ascorbate.

On the other hand, we did not find any significant difference in SOD activity with RVT pretreatment. SOD catalyzes the dismutation of O_2_^−^ into oxygen and hydrogen peroxide. The lack of difference with and without RVT pretreatment may be due to the fact that the pro-oxidant ferrous iron/ascorbate generates the hydroxyl radical through the Haber-Weiss or Fenton reaction [40]. 

Oxidative damage is considered the main indicator of a loss of cellular function caused by oxidative stress [1]. Hence, evaluation of the TBARS concentration was carried out with the four groups of the present study. The results indicate that there is oxidative damage to spermatozoa membranes caused by ferrous iron/ascorbate, as well as protection against such damage by RVT pretreatment. In accordance with these results, previous studies have reported that RVT prevents the lipid peroxidation induced by tert-butyl hydroperoxide in human sperm [45], and that RVT is a lipophilic molecule that prevents lipid peroxidation induced by Fenton reaction products [44,46]. The protective effects of RVT against oxidative damage might be attributed to a hydrogen electron donation from its hydroxyl groups [47].

To further explore the protective role of RVT against the oxidative stress in spermatozoa caused by ferrous iron/ascorbate, we evaluated the IVF capacity of the four groups, finding that RVT pretreatment protected the IVF process. The results of various reports are consistent with this observation. For example, it has been reported that the oxidative damage induced by ferrous iron/ascorbate leads to a decrease in cholesterol efflux in macrophages, and that this decrease is restored by RVT treatment in a concentration-dependent manner [46]. It is also known that enhanced cholesterol efflux is involved in improving sperm capacitation and IVF capacity [48]. One antioxidant, glutathione, has recently been shown to improve the IVF process by reducing ROS production and peroxidative damage in the acrosome [49]. The results of two recent studies support this same role of RVT in the protection of IVF capacity. The antioxidant potential of RVT (to reduce the level of ROS and increase the level of glutathione) has been used to improve the maturation of spermatozoa and the IVF process in porcine oocytes [50]. Additionally, RVT has been shown to have a beneficial effect on the development of porcine embryos [51].

## 5. Conclusions

In conclusion, the present study provides evidence that RVT is a potent antioxidant *in vitro*. RVT was shown to protect spermatozoa from oxidative damage caused by ferrous iron/ascorbate through protection from lipid peroxidation and preservation of the IVF process.
